# Picture quiz

**Published:** 2018-02-08

**The chart below shows the number of blind people per million population for the world, and different regions, between 1990 and 2015.**

**Figure:**
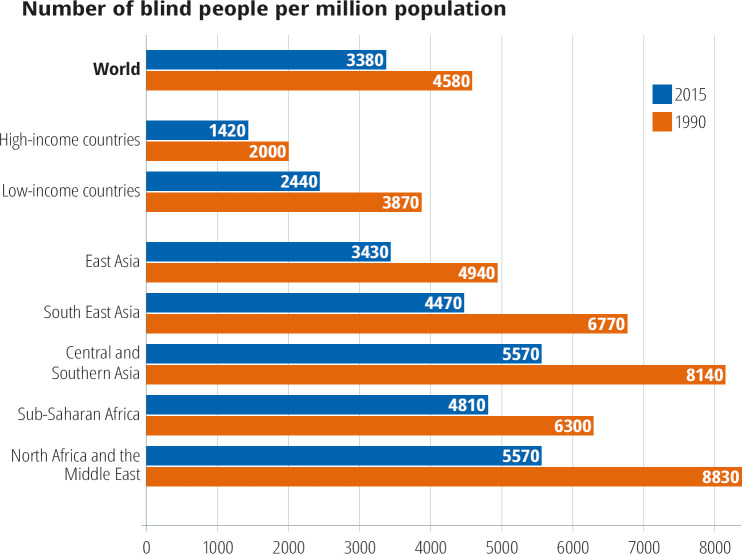

**Question 1 What two trends are shown in the figure?**
**Question 2 What is the ratio of blindness in 2015 between sub-Saharan African countries and high-income countries?**
**Question 3 Why do low-income countries have more blindness per million population than high-income countries?**
**Question 4 Why might you expect high-income countries to have more blindness per million population than low-income countries?**

## ANSWERS

1. Decrease in the number of blind/ million population between 1990 and 2015 in all regions; and decrease in the number of blind in regions with better economies (High income countries, compared to Asia, compared to Africa).
2. 4810/1480 blind people per million pop; counties in SSA have 3.25 times more blind people than high income countries.
3. Low income countries have less resources for health care – health trained staff and facilities so less eye care services.
  Also low income countries have greater levels of poverty, so fewer people can afford to pay for health services.
  People living in low income countries have greater exposure to some risk factors that cause eye diseases e.g. poor diet; poor water and sanitation etc.
4. High income countries have older populations so there are more people over the age of 50 years in each million population. Because visual impairment and blindness are associated with aging one would therefore expect more blindness per million population in countries with older populations.

